# Impact of review of histopathology specimens at a tertiary oncology hospital in Eastern India—lessons learnt

**DOI:** 10.3332/ecancer.2022.1441

**Published:** 2022-08-25

**Authors:** Debdeep Dey, Bhagat Singh Lali, Paromita Roy, Divya Midha, Indu Arun, Lateef Zameer, Anand Bardia, Geetashree Mukherjee

**Affiliations:** Department of Oncopathology, Tata Medical Center, 14, MAR(E-W), DH Block(Newtown), Action Area I, Newtown, Kolkata, West Bengal 700160, India

**Keywords:** oncology, histopathology, diagnosis, concordance, discrepancy

## Abstract

A central review of histopathology specimens at tertiary oncology hospitals is important for optimum patient care in the modern era of personalised medicine. The challenges of healthcare delivery and access to ancillary investigations faced by a pathologist from the Indian subcontinent are different from the western world. We undertook an audit to analyse the differences of opinion between the diagnosis offered at peripheral hospitals and a tertiary oncology hospital in Eastern India. By analysing the differences, common pitfalls and diagnostic discrepancies are identified which need to be addressed in future. This audit also highlights the need of setting up of tertiary oncology diagnostic centres to help both peripheral pathologists and cancer care clinicians like a hub and spoke model. This is most needed for haematopathology, soft tissue and gynaecologic oncology where the need of ancillary investigations is high.

## Introduction

Histopathology review of specimens from patients who seek cancer care at a tertiary oncology hospital constitutes a significant proportion of the routine workload for pathologists. While most of these reviews or second opinions are generated as part of institutional policy, some cases are also patient driven or a pathologist seeking a second opinion. A literature search reveals articles mainly from the western world [1–3] about the impact of review of the histopathology specimens on patient management; however, there is a paucity of literature from the Indian subcontinent [4] regarding the importance of such reviews. This is essential and pertinent in view of the differences in the challenges of healthcare system and access to ancillary investigations faced by pathologists of the Indian subcontinent as compared to their counterparts from the Western world. Being a tertiary oncology centre of repute, we receive a significant number of such reviews from peripheral hospitals and laboratories wherein slides/paraffin blocks and a copy of histopathology report are submitted for analysis. This review process becomes an integral part of patient management as the treatment is initiated after confirmation of the diagnosis rendered at the peripheral diagnostic centres.

This audit aims to analyse the differences of opinion between the diagnosis offered at a peripheral hospital/laboratory and our hospital with respect to the concordance rates, major and minor discordant results and their impact on patient management. Further analysing the differences, major diagnostic discrepancies are highlighted to pinpoint the potential challenging areas in the field of pathology.

## Methods

We undertook an institution based observational descriptive study wherein all the cases received for second opinion/review from January to December 2021 were included. These cases were received at our centre as part of institutional policy to review the histopathology slides and/or blocks for final diagnosis before treatment is initiated. Cases where the tissue was inadequate for opinion due to depleted tissue in the paraffin block or those where additional tissue was required to give the complete diagnosis were excluded from the study. The outside diagnosis and final diagnosis rendered by us was correlated and results were divided into four categories: concordance, major discordance, minor discordance or refinement. Major discordance was defined as the one which may lead to serious errors in clinical management and treatment (for example, benign versus malignant, incorrect histogenesis of the tumour or even incorrect grading of Non-Hodgkin’s lymphoma). Every major discordant diagnosis was reviewed and confirmed by another intra-departmental colleague to ensure conformity (third pathologic review). Minor discordance was defined as one where the patient management and treatment is not affected (for example, differences in tumour sub classification that did not affect treatment). Refinement was defined as cases where the diagnosis was made more precise (for example, a diagnosis of lymphoproliferative disorder further refined as follicular lymphoma, grade 2 on review). The various result categories were further analysed with respect to the field/subspeciality of pathology. Results were entered and stored in our laboratory reporting system (AnPath software) and made available on the patient electronic medical records for the treating team to tailor further therapy. These results were also entered in Microsoft Excel as part of data collection and further analysed by the pathologist.

## Results

In total, 700 cases were included in the present study, as per the inclusion and exclusion criteria. Suspected or diagnosed neoplastic disease process was the major cause of review in 100% of the cases. On detailed analysis, concordant diagnosis was offered in 446/700 (64%) of cases. Among the disagreements, a major discordant diagnosis was made in 117/700 (17%) of the cases, a minor discordant diagnosis was offered in 34 (5%) of the cases and refinement of the diagnosis was done in 103 (14%) of the cases. The three most common subspecialities where the results had major discordance were haematolymphoid system (23%) followed by gynaecologic pathology (18%) and skin and soft tissue pathology (15%). Minor discordance was observed most frequently in cases from gastrointestinal pathology (29%) followed by gynaecologic pathology (23%) and head and neck pathology (21%) (Table 1).

A major error of benign versus malignant diagnosis was observed in 11 haematolymphoid cases, 11 skin and soft tissue cases, 10 head and neck cases, 5 gynaecology cases, 4 thoracic cases and 3 urology cases. Significant incorrect sub typing of the tumour was observed in 16 haematolymphoid cases, 16 gynaecology cases, 7 skin and soft tissue cases, 7 thoracic cases, 6 urology cases, 5 breast cases, 4 gastrointestinal cases and 3 head and neck cases (Tables 2–4; Supplementary Tables S1–S5) These major discordant results significantly affected the patient treatment and management.

## Discussion

In our audit, out of the 700 cases, 64% had a concordant diagnosis. This is lower than the concordance rates reported by Middleton *et al* [1] from MD Anderson Cancer Center (75% concordance) or Strosberg *et al* [2] from the University of South Florida (85% concordance). Similarly, Farooq *et al* [5] reported 3.7% cases with major discordance without any change in management and 1% major discordant cases with clinical change in management on pathologic review at the Medical College of Wisconsin and Johnson *et al* [6] reported 4.1% cases wherein patient care was changed after pathologic review at their institute. The major discordant results were reported in higher numbers in our audit (117 cases, i.e. 17%) as compared to Middleton *et al* [1] (6.3%) and Strosberg *et al* [2] (2.2%). The higher percentage of discordance, mainly major discordance, in our study reflects a wide variation in the quality of diagnostic services available in this part of the world and is a cause of concern for patient mismanagement in significantly higher numbers (117 patients in our audit).

While Middleton *et al* [1] have reported the highest discordance rates among head and neck pathology cases (46%) and Strosberg *et al* [2] reported it in neuropathology cases (10.9%), we found major discordance in haematolymphoid, gynaecologic and skin and soft tissue cases. These variations represent the varying levels of experience with respect to certain sub-specialities among the pathologists in the outside centres and also on the distribution of workload among them.

### Haematolymphoid system

We found maximum major discordance among haematolymphoid cases (23%). Laurent *et al* [7] have reported significant impact on patient care in 17.4% of cases reviewed by an expert. This is expected as not only the speciality is challenging with rapidly emerging entities but ancillary investigations, including immunohistochemistry and molecular testing, form an integral part of the diagnostic work-up and such facilities are not available in many peripheral laboratories or hospitals.

Disagreement between benign versus malignant diagnosis within haematolymphoid system was high. Careful assessment of the lymph node architecture with special attention to extra capsular spread and correlation with clinical details, like immunosuppressant or transplant, are key to an accurate diagnosis [8]. Notable reactive conditions which mimicked neoplasm were autoimmune diseases and methotrexate associated lymphoproliferative disorder. Nodular lymphocyte predominant Hodgkin lymphoma is a neoplastic entity which was frequently diagnosed as reactive process. This is because the popcorn cells can easily be missed if the sections are sub optimally processed and/or stained. Similarly, classical Hodgkin lymphoma was diagnosed as a chronic fibrosing process. This is particularly true for the nodular sclerosis variant which can be mistaken for fibrosis. Therefore, every fibrotic appearing histology in a lymph node or mediastinal lesion warrants CD30 immunotesting to confirm or exclude Hodgkin lymphoma.

**Figure 1.**
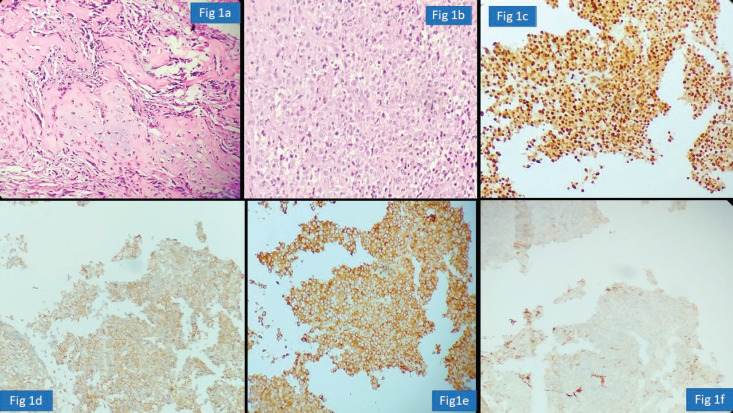
Plasma cell neoplasm misdiagnosed as Chondrosarcoma (a): Haematoxylin-eosin (HE)×100; exuberant fracture callus formation (b): HE ×100; sheets of atypical plasma cells with binucleate forms. (c): MUM1 positive cells. (d): CD138 positive. (e): Kappa. (f): Lambda.

In our audit, plasma cell neoplasms were misdiagnosed as round cell sarcoma or malignant round cell tumour. A correct diagnosis needs careful analysis of the cells which classically display eccentric nuclei, thus ordering the requisite immunostains such as CD138 and Multiple Myeloma 1 (MUM1) (Figure 1a–f). Some cases involved incorrect histogenesis of the tumour (such as ALK negative ALCL misdiagnosed as myoepithelial carcinoma). These entities need high index of suspicion and detailed immunohistochemistry to render a correct diagnosis.

The pathologists should be aware of the rare diagnosis such as ALK positive large B-cell lymphoma, plasmablastic lymphoma and Rosai Dorfman disease in order to request requisite investigations. The correct diagnosis is rendered on expert consultation where the expert pathologist has continuous exposure to such cases and access to multiple immunohistochemical stains. Therefore, one must not refrain from communicating the diagnostic dilemma in the final report with recommendation of getting an expert review prior to initiation of therapy.

Some cases involved incorrect grading of lymphoma such as DLBCL misdiagnosed as marginal zone lymphoma and B-ALL misdiagnosed as low-grade B-cell lymphoma. Careful attention to the cytological details and correlation with TdT and/or Ki-67 proliferation index helps to prevent such misdiagnosis [8]. A note of caution here is that a sub optimally fixed specimen will have lower Ki-67 and small size of the cells making it difficult to recognise a high-grade lymphoma.

### Gynaecology

Gynaecology cases constituted 18% of the major discordant diagnosis in our audit. Eskander *et al* [9] have reported major discordant diagnosis in 6.8% of reviewed cases of gynae-oncology. Most of the discordant diagnosis attributed to the typing of carcinomas. This has major consequences in patient management as the extent of staging surgery and adjuvant therapy, including targeted therapy, differs significantly as per the subtype of carcinoma [10].

The misdiagnosis between endometrioid and serous carcinoma was frequently encountered. Careful attention to cytological details, architecture (papillary, glandular, cribriform with slit like fenestrations) and a comprehensive Immunohistochemistry (IHC) panel of p53, p16, ER and Wilms tumour suppressor gene 1 (WT-1) help in precise diagnosis. Tumour protein 53 immunostain is wild type in endometrioid carcinoma while it is mutated (diffuse block positive or null type staining) in serous carcinomas. It is important to identify high-grade adenocarcinomas (clear cell carcinoma, serous carcinoma and high-grade endometrioid carcinoma) for relevant and effective treatment [11].

Next common misdiagnosis was distinction between endometrial endometrioid carcinoma and adenocarcinoma of cervix. In this scenario, the panel of Progesterone receptor (PR) & vimentin (both positive in endometrioid carcinoma) with p16, Carcinoembryonic antigen (positive in endocervical carcinoma) is very helpful. In few cases where immunohistochemistry is inconclusive, Human Papilloma Virus-DNA testing helps to differentiate between them.

**Figure 2.**
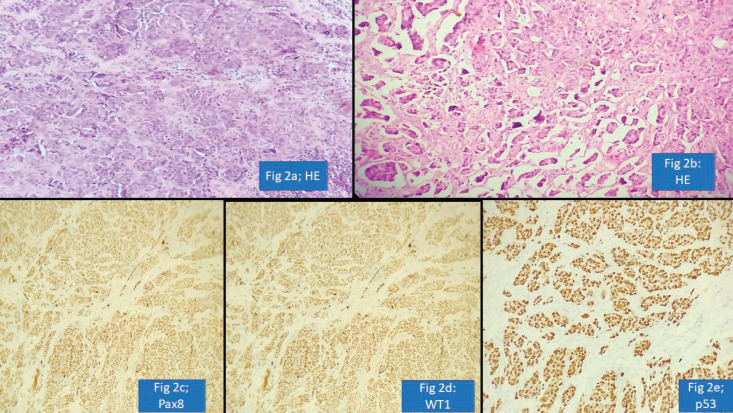
Appendiceal involvement by Serous Carcinoma misdiagnosed as Neuroendocrine tumour (a): HE ×100; nests of tumour cells. (b): HE ×100; nests of tumour cells with retraction artefact. (c): PAX-8 positive cells. (d): WT-1 positive cells. (e): p53 shows strong and diffuse nuclear staining.

We observed another diagnostic challenge of inability to identify gynaecological origin of the tumour when the biopsied organ was non-gynaecological such as lung, bladder and appendix. This has huge ramification as very often this would be the first organ where the diagnosis will be made and thus, treatment protocols might vary significantly. For instance, an appendiceal tumour misdiagnosed as neuroendocrine tumour was rendered a correct diagnosis of high-grade serous carcinoma which was the initial presenting symptom in a female patient (Figure 2a–e) Inadequate tissue sampling or submission of only few slides/blocks from the tumour often lead to misdiagnosis in ovarian tumours. Adequate sampling of ovarian tumour is essential as a small focus of invasive carcinoma or mural nodule of anaplastic carcinoma in otherwise borderline tumour has major treatment implications. It is essential for the pathologist to study two sections for every centimetre of an ovarian tumour which has a heterogenous or solid cystic appearance [12].

### Soft tissue and skin

Skin and soft tissue cases constituted 18/117 (16%) of the major discordant diagnosis. Ray-Coquard *et al* [13] reported major discordance in 8% cases. Thway *et al* [14, 15] have reported a major diagnostic discrepancy in 10.9% soft tissue tumour cases without molecular testing and 16.4% cases in era of molecular testing.

A precise diagnosis of sarcomas requires optimally stained tissue sections, immunohistochemistry and molecular studies. Continuous exposure of the reporting pathologist to various entities is essential. Lower incidence of soft tissue sarcomas and wide variation in classification systems necessitate an expert review in most cases. Site wise correlation of the histopathology findings with smart IHC work-up is the key to diagnosis. For instance, a biopsy from L5-S1 region needs careful morphological analysis for physaliferous cells along with brachyury immunohistochemistry to differentiate chordoma from metastatic carcinoma. The diagnosis of osteosarcoma would require mandatory correlation with radiology. Synovial sarcoma and PNET require not only immunohistochemistry but fluorescence in situ hybridisation testing; however, the pathologist at the peripheral laboratory/hospital should mention the need for a second opinion in the report so that precise diagnosis is established prior to initiation of treatment.

In skin tumours, most frequent diagnostic discrepancy was encountered in pigmented or melanocytic lesions. This is similar to results from Italian authors who found diagnostic discrepancies in whopping 56% cases of melanomas and relevant treatment changes in 27.3% cases [16]. An aneurysmal fibrous histiocytoma was misdiagnosed as malignant melanoma with a pT4 stage. Basic Perl’s stain to identify the brown pigment as iron and not melanin would help in making this diagnosis. There is a spectrum of melanocytic lesions and the pathologist needs to be conversant with the varieties of melanocytic lesions like lentigo simplex and melanocytic acral naevus for accurate diagnosis.

Another common diagnostic discrepancy was between skin adnexal tumours and squamous cell carcinoma. A long clinical history with sudden spurt in growth is often the first clinical clue that this could be an adnexal tumour. Thus, critical analysis of clinical history with morphological analysis always helps to render correct diagnosis of cutaneous adnexal neoplasms.

### Urological pathology

**Figure 3.**
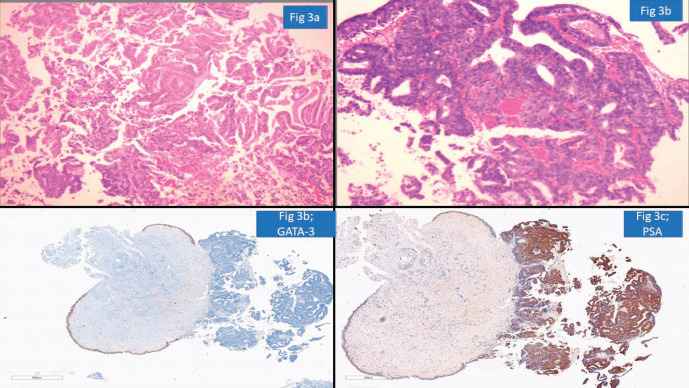
Prostatic adenocarcinoma involving urinary bladder misinterpreted as Urothelial carcinoma (a): HE ×100-glandular morphology. (b): glands are composed of cells with moderate nuclear pleomorphism and complex architecture. (c): GATA-3 negative in tumour with positive internal control. (d): PSA positive suggesting prostatic origin.

Urology cases constituted 15/117 (13%) of the major discordant diagnosis affecting patient treatment. Chen *et al* [17] reported potential for change in treatment in 9.3% prostatic cancer patients on second review of prior biopsy. We observed frequent major disagreement between establishing the diagnosis of urothelial carcinoma or prostatic adenocarcinoma particularly in high-grade poorly differentiated tumours. Vigilant search for surface urothelial dysplasia and detailed morphological analysis coupled with the use of GATA binding protein 3 (GATA-3), Prostate specific antigen (PSA) and NK3 homeobox-1 helps in arriving at the correct diagnosis [18]. The pathologist should work up the differential diagnosis of prostatic adenocarcinoma, particularly ductal type, when one encounters a glandular morphology in a bladder biopsy (Figure 3a–d). A metastasis or direct involvement by serous carcinoma or cervical squamous cell carcinomas in females should also be considered and requisite IHC be done. These scenarios have major therapeutic and prognostic implications.

In prostatic adenocarcinoma Gleason score 5, one should always consider differential diagnosis of lymphoma and small cell carcinoma. Even in garden variety prostatic adenocarcinoma, it is always worthwhile looking for a neuroendocrine carcinoma component morphologically as these tumours are likely to behave more aggressively [3].

We also encountered other major discordant results, namely germ cell tumours misdiagnosed as metastatic adenocarcinoma, overdiagnosis of muscularis propria invasion in transurethral resection of bladder tumours, grading of urothelial carcinomas and major disagreements in Gleason scoring of prostatic adenocarcinomas.

### Gastrointestinal pathology

**Figure 4.**
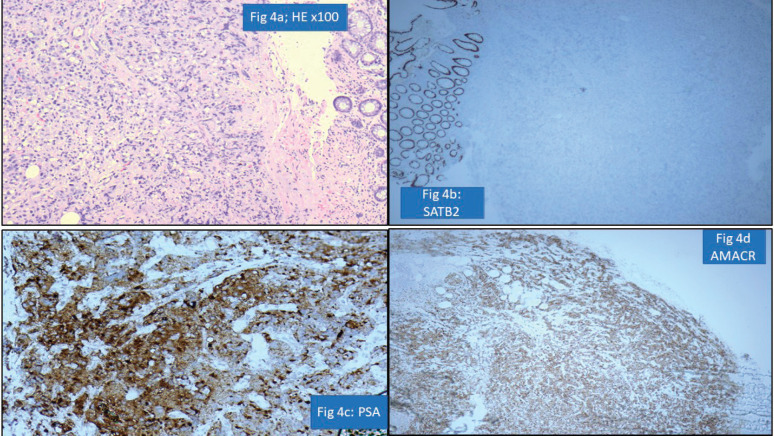
Prostatic adenocarcinoma involving rectum misinterpreted as Rectal adenocarcinoma (a): HE ×100-poorly differentiated submucosal tumour in rectum. (b): SATB2 negative in tumour cells with positive internal control. (c): PSA positive tumour cells. (d): AMACR positive tumour cells.

One of the important areas of discordance was metastatic tumours to the gastrointestinal tract, particularly in rectal and sigmoid colon biopsies where involvement by prostatic carcinomas and serous carcinomas is a possibility. Particular attention to surface epithelial dysplasia and tumour morphology (dirty necrosis in the colonic malignancies, cribriform glandular pattern in prostatic malignancies, high-grade cytology of serous carcinoma) along with the use of immunohistochemistry like PSA, SATB homeobox-2 (SATB2) and paired box gene 8 always aids in precise diagnosis [19]. For instance, a rectal biopsy with submucosal location of a poorly differentiated tumour was diagnosed as metastatic prostatic adenocarcinoma as the tumour cells were negative for SATB2 but positive for PSA and Alpha Methyacyl CoA racemase (AMACR) (Figure 4a–d).

Entities such as neuroendocrine tumours and metastatic adenocarcinoma of stomach need to be suspected morphologically and further subjected to requisite immunohistochemistry to confirm the diagnosis as treatment protocols and outcomes vary significantly.

Discordance was seen with respect to mixed adenoneuroendocrine carcinomas where neuroendocrine component was often missed. Another area of minor discordance was in the grading of dysplasia.

Head and neck pathology

Head and neck pathology cases contributed 13/117 (11%) of the major discordant diagnosis. Similarly, Zhu *et al* [20] reported a whopping overall discordance of 22% in their study on head and neck pathology second review with histology being the most common cause of disagreement. A major discrepancy was related to squamous dysplasia and invasive carcinomas. The diagnoses of these lesions need assessment of surface dysplasia (present in squamous cell carcinoma) and the broad bulbous tongues of squamous epithelium with a pushing edge coupled with radiological correlation helps in clinching the diagnosis. Sometimes it is possible that a definite diagnosis cannot be offered in a small biopsy where a broad diagnostic terminology like atypical squamoproliferative lesion should be used which will help the clinician in taking the next clinical step.

Significant discordance was also encountered in histological diagnosis of thyroid tumours. For instance, follicular variant of papillary thyroid carcinoma is misdiagnosed as follicular carcinoma. Detailed study of the nuclear details like nuclear irregularity, grooving, clearing, overlapping and microfollicular pattern is helpful in rendering the correct diagnosis. On the other hand, whenever the cells have plasmacytoid or spindle cell morphology with amyloid deposition, diagnosis of medullary thyroid carcinoma should be considered. Misdiagnosis can have major therapeutic and prognostic consequences affecting patient care.

### Lung/thoracic pathology

We found major discordance in 10% of the thoracic pathology cases reviewed at our centre. Similarly, Farooq *et al* [5] have reported discordance in 3% of thoracic biopsies reviewed. One of the major patterns of discordance revealed was in the typing of carcinomas – including broad misdiagnosis between small cell and non-small cell carcinoma. The diagnosis of squamous cell carcinoma is clinched on morphological findings of keratinisation, intercellular bridges and/or immunohistochemistry with p40. On similar grounds, gland formation, identification of mucin in at least five tumour cells by special stains such as Alcian blue and/or immunopositivity with Thyroid transcription factor 1 (TTF-1) enables the correct diagnosis of adenocarcinoma. While nuclear moulding, inconspicuous nucleoli and brisk mitotic activity along with positive neuroendocrine markers help in the diagnosis of small cell carcinoma, a note of caution is TTF-1 can be positive in both small cell carcinoma and adenocarcinoma [21]. Hence, merely relying on TTF-1 without attention to morphology often misleads to a wrong diagnosis. Similarly, a metastasis of thyroid carcinoma in lung would not get picked up if there is too much reliance on TTF-1 without careful attention to morphology. In such cases, thyroglobulin and PAX-8 immunostains would be helpful. Other cases of minor discordance were in typing of non-small cell carcinomas where the final diagnosis was refined.

### Breast pathology

Breast pathology cases comprised 5/117 (4%) of the major discordant diagnosis. Soofi and Khoury [22] have reported a major discordance in 8% cases reviewed at their centre with major treatment implications. In terms of epithelial malignancies, the major discrepancies were in typing of carcinomas. Invasive focus was missed in few cases including invasive papillary carcinoma where the diagnosis is based on morphology and breast panel immunohistochemistry (Estrogen receptor (ER), PR, Human epidermal growth factor receptor 2). Other misdiagnosed cases belonged to spectrum of mesenchymal lesions where attention to morphology and mitotic figures would enable a correct diagnosis in most cases.

The minor discordant diagnoses were in typing of carcinoma (like invasive ductal and invasive lobular carcinoma and solid papillary carcinoma).

### Lessons learnt

1. The audit revealed substantial number of cases with suboptimal fixation and lack of good H&E stain which made the job harder for the pathologist. Suboptimal fixation shrinks the cell size with lower Ki-67 proliferation index making correct grading of lymphomas, neuroendocrine tumours and sarcomas difficult.
2. If the morphology and immunohistochemistry do not fit into a carcinoma of a particular organ, always consider a metastasis from other sites.
3. Aberrant expression of immunohistochemistry is a potential diagnostic pitfall which one needs to be aware of. For example, plasma cell neoplasms expressing CD20, plasmablastic lymphomas expressing CD3 and peripheral T-cell lymphomas expressing CD20 could lead to major diagnostic errors.
4. Extensive sampling of tumours of the ovary (particularly borderline tumours) or sarcomas is mandatory as it can reveal focal morphological detail which clinches the diagnosis.
5. Histopathology needs to be interpreted in proper clinical and radiological correlation for meaningful results. Mutual communication with the treating team always proves beneficial for optimum patient care.
6. It is important to mention the diagnostic dilemma or uncertainty in the diagnosis when the ancillary investigations are not available. Terms such as lymphoproliferative disorder or high-grade adenocarcinoma would be acceptable from a peripheral hospital prompting the treating clinician to chase a definite diagnosis by review at a tertiary centre.
7. Subspeciality reporting significantly improves diagnostic accuracy which is very essential in today’s era of personalised medicine in oncology care. Continuous exposure to rare diagnosis refines the diagnostic accuracy.

## Conclusions

The current audit highlights the need of consolidation of histopathology services by setting up of tertiary diagnostic centres which would feed from the peripheral laboratories or hospitals. In the dynamic era of modern pathology, histopathology needs constant exposure to the different lesions for easy recognition. This highlights the need for capacity building to ensure regular exposure of general pathologists to such case scenarios in structured training programmes from time to time. This is particularly true for haematolymphoid and soft tissue pathology where the multiple diagnostic entities are overlapping and the need of ancillary investigations is high. Even after untiring efforts, if there is a diagnostic dilemma, it must be clearly stated in the histopathology report and a second review recommended in the best interests of the patient.

## List of abbreviations

DLBCL, Diffuse large B cell lymphoma; ALCL, Anaplastic large cell lymphoma; PNET, Primitive neuroectodermal tumour; DCIS, Ductal carcinoma in-situ; DFSP, Dermatofibrosarcoma protuberans.

## Declarations

### Ethical consideration

This is a retrospective audit with no disclosure of personal details so no informed consent was needed.

### Conflicts of interest

- The authors have no relevant financial or non-financial interests to disclose.
- The authors have no competing interests to declare that are relevant to the content of this article.
- All authors certify that they have no affiliations with or involvement in any organisation or entity with any financial interest or non-financial interest in the subject matter or materials discussed in this manuscript.
- The authors have no financial or proprietary interests in any material discussed in this article.

### Funding

No funding was received or required for the preparation of this piece.

### Ethics and patient consent

The information presented in this publication has no personally identifiable material. Neither were any interventions or changes in management done for this publication. This study has not posed, and does not pose, any risk to the patient concerned. Thus, a waiver of consent is justified in this case.

### Author contributions

Dr Dey designed the audit and did the data collection. Dr Dey and Dr Lali prepared the manuscripts. All authors have reviewed the cases, read and approved the manuscript.

## Figures and Tables

**Supplementary Table S1.** Major discordant diagnosis in urology.

Prior to review	Diagnosis after review
**A) Benign/malignant discrepancy**	
Polypoid clear cell lesion	Metastatic renal cell carcinoma
Negative for malignancy	Adenocarcinoma of prostate
Squamous cell carcinoma	Radiation induced atypia
**B) Incorrect typing of tumour**	
Bladder transitional cell carcinoma	Prostatic adenocarcinoma, ductal type
Prostatic adenocarcinoma (2)	Urothelial carcinoma (2)
Metastatic adenocarcinoma	Germ cell tumour
Low-grade urothelial carcinoma (5)	High grade urothelial carcinoma (5)
**C) Incorrect histogenesis of the tumour**	
Adenocarcinoma	Adenocarcinoma & small cell carcinoma
Adenocarcinoma Gleason score 5	High grade lymphoma

**Supplementary Table S2.** Major discordant diagnosis in gastrointestinal system.

Prior to review	Diagnosis after review
**A) Benign/malignant discrepancy**	
Plasma cell neoplasm	Inflammatory lesion
**B) Incorrect typing of tumour**	
Rectal adenocarcinoma (2)	Prostatic adenocarcinoma (2)
Mesenchymal neoplasm	Well differentiated neuroendocrine tumour
Serous carcinoma	Metastatic adenocarcinoma from stomach
Adenocarcinoma	Tubulovillous adenoma

**Supplementary Table S3.** Major discordant diagnosis in head and neck pathology.

Prior to review	Diagnosis after review
**A) Benign/malignant discrepancy**	
Verrucous carcinoma	Hyperplastic squamous epithelium
Inflammatory myofibroblastic tumour	Inflammatory fibrocollagenous tissue
High-grade mucoepidermoid carcinoma	Pleomorphic adenoma
Colloid nodule	Papillary thyroid carcinoma
Colloid goitre	Malignant spindle cell neoplasm
Sinonasal carcinoma	Meningioma
Adenocarcinoma	Pleomorphic adenoma
Metastatic squamous cell carcinoma	Granulomatous lymphadenitis￼
Papillary thyroid carcinoma	Negative for malignancy
Verrucous hyperplasia	Squamous cell carcinoma
**B) Incorrect typing of the tumour**	
Follicular adenoma	Follicular variant of papillary thyroid carcinoma
Metastatic adenocarcinoma	Metastatic medullary thyroid carcinoma
Squamous cell carcinoma	Small cell carcinoma

**Supplementary Table S4.** Major discordant diagnosis in thoracic pathology.

Prior to review	Diagnosis after review
**A) Benign/malignant discrepancy**	
Small cell carcinoma	Chronic inflammation
Sarcoma	Neurofibroma
Adenocarcinoma	Negative for malignancy
Squamous cell carcinoma	Negative for malignancy
**B) Incorrect typing of tumour**	
Squamous cell carcinoma	Adenocarcinoma
Squamous cell carcinoma	Small cell carcinoma
Adenocarcinoma/pleomorphic adenoma	Metastasis of papillary thyroid carcinoma
Adenocarcinoma	Small cell carcinoma
Metastatic adenocarcinoma	Metastatic small cell carcinoma
Adenocarcinoma	Small cell carcinoma
Squamous cell carcinoma	Adenocarcinoma

**Supplementary Table S5.** Major discordant diagnosis in breast pathology.

Prior to review	Diagnosis after review
**A) Incorrect typing of tumour**	
Invasive carcinoma with no lymph node metastasis	Invasive carcinoma with lymph node metastasis
**Encapsulated with small invasive**	**Invasive papillary carcinoma**
Ductal carcinoma in-situ (DCIS)	DCIS with invasive
DFSP	Phyllodes tumour
Phyllodes borderline tumour	Malignant phyllodes tumour

**Table 1.** Subspeciality wise distribution of the major discordant, minor discordant and refinement results.

**Major discordance**		
Haematolymphoid	27	23%
Gynae-oncology	21	18%
Skin and soft tissue	18	15%
Head & neck	13	11%
Urology	15	13%
Thoracic	11	10%
Breast	6	5%
Gastrointestinal	6	5%
**Minor discordance**		
Gastrointestinal	10	29%
Gynae-oncology	8	23%
Head & neck	7	21%
Breast	5	15%
Haematolymphoid	2	6%
Thoracic	1	3%
Soft tissue	1	3%
**Refinement**		
Haematolymphoid	36	35%
Gynae-oncology	25	24%
Thoracic	10	9%
Head & neck	8	8%
Gastrointestinal	8	8%
Urology	6	6%
Soft tissue	5	5%
Breast	5	5%

**Table 2.** Major discordant diagnosis offered in haematolymphoid system.

Prior to review	Diagnosis after review
A) Benign/malignant discrepancy	
Diffuse large B-cell lymphoma (DLBCL)	Methotrexate associated lymphoproliferative disorder
Granulomatous inflammation	Nodular lymphocyte predominant Hodgkin lymphoma with granulomas
Reactive lymphadenopathy	Nodular lymphocyte predominant Hodgkin lymphoma
Rosai Dorfman disease	DLBCL
Epstein-Barr virus (EBV) positive DLBCL	Immunoblastic proliferation with autoimmune diseases
Reactive lymphadenopathy	DLBCL
Reactive lymphadenopathy	Lymphoproliferative disorder
Hodgkin lymphoma	Reactive lymphadenopathy
Lupus panniculitis	Subcutaneous panniculitis type T-cell lymphoma
Metastatic squamous cell carcinoma	Granulomatous lymphadenitis
Chronic fibrosing process	Hodgkin lymphoma
B) Incorrect typing of lymphoma	
Low-grade Non-Hodgkin lymphoma (NHL)	B cell Acute Lymphoblastic Leukaemia (B-ALL)
Marginal zone lymphoma	DLBCL
Hodgkin lymphoma	Follicular lymphoma
DLBCL	Plasmablastic lymphoma
Marginal zone lymphoma	DLBCL
T cell lymphoma	High-grade B cell NHL
Hodgkin lymphoma	ALK+ large B cell lymphoma
Non-Hodgkin lymphoma	Classical Hodgkin disease
Hodgkin lymphoma	T cell rich large B cell lymphoma
C) Incorrect histogenesis of the tumour	
Myoepithelial carcinoma	Anaplastic large cell lymphoma (ALCL), Anaplastic Lymphoma Kinase (ALK) negative
Hodgkin lymphoma	Undifferentiated carcinoma
Adenocarcinoma	High-grade B-cell non-Hodgkin lymphoma
Malignant round cell tumour	Plasma cell neoplasm
Inflammatory lesion	Rosai Dorfman disease
Osteo/chondrosarcoma	Plasma cell neoplasm
Round cell sarcoma	Plasma cell neoplasm

**Table 3.** Major discordant diagnosis in gynae-oncology system.

Prior to review	Diagnosis after review
A) Benign/malignant discrepancy	
Ulceration	Differentiated vaginal intraepithelial neoplasia
Leiomyoma	Endometrial stromal sarcoma
Leiomyoma	Epithelioid leiomyosarcoma
Negative for malignancy	Serous carcinoma
Low-grade serous surface papillary adenocarcinoma	Serous cystadenoma with focal epithelial proliferation
B) Incorrect typing of tumour	
Adenocarcinoma of endometrium	Squamous cell carcinoma of cervix
Serous papillary carcinoma	Endometrioid carcinoma
Endometrioid carcinoma	Endometrioid and clear cell carcinoma
Endometrioid carcinoma	High-grade serous carcinoma
Adenocarcinoma	High-grade serous carcinoma
Lung adenocarcinoma	Metastasis of serous carcinoma
Endometrial adenocarcinoma	High-grade serous carcinoma
High-grade serous carcinoma	Small cell carcinoma of hypercalcaemic type
Borderline mucinous tumour	Mucinous carcinoma with mural nodule of anaplastic carcinoma
Adenocarcinoma of bladder	High-grade serous carcinoma of Mullerian tract
Endometrioid carcinoma	High-grade serous carcinoma
Endometrioid carcinoma	Adenosquamous carcinoma of cervix
Squamous cell carcinoma	Cervical Intraepithelial neoplasia 3 with involvement of endocervical crypts
C) Incorrect histogenesis of the tumour	
Low-grade Endometrial stromal sarcoma	Invasive squamous cell carcinoma
Pleomorphic sarcoma	Malignant melanoma
Neuroendocrine tumour	High-grade serous carcinoma

**Table 4.** Major discordant diagnosis in skin and soft tissue.

Prior to review	Diagnosis after review
A) Benign/malignant discrepancy	
Malignant melanoma	Aneurysmal fibrous histiocytoma
Squamous cell carcinoma	Proliferating trichilemmal cyst
Fibrous dysplasia	Osteosarcoma
Squamous cell carcinoma	Hidradenoma
Angiofibroma	Osteosarcoma
Basal cell carcinoma	Eccrine poroma
Malignant melanoma	Lentigo simplex
Cutaneous lymphoma	Lichenoid dermatitis
Squamous cell carcinoma	Hypertrophic lichen planus
Squamous cell carcinoma	Eccrine poroma
Dermatofibrosarcoma protuberans (DFSP)	Dermatofibroma
B) Incorrect typing of tumour	
Metastatic carcinoma	Chordoma
Benign hyperkeratotic lesion	Atypical melanocytic lesion
Gastrointestinal; stromal tumour	Dedifferentiated liposarcoma
Chondrosarcoma	Low-grade sarcoma
C) Incorrect histogenesis of the tumour	
Neuroendocrine tumour	Synovial sarcoma
Dysgerminoma	Primitive neuroectodermal tumour (PNET)
Fibromatosis	Sarcomatoid carcinoma
